# Aflatoxins in rice: Worldwide occurrence and public health perspectives

**DOI:** 10.1016/j.toxrep.2019.11.007

**Published:** 2019-11-05

**Authors:** Nurshad Ali

**Affiliations:** Department of Biochemistry and Molecular Biology, Shahjalal University of Science and Technology, Sylhet 3114, Bangladesh

**Keywords:** Aflatoxins, Rice, Occurrence, Biomarker, Exposure, Human health

## Abstract

Aflatoxins are fungal secondary metabolites that contaminate dietary staples worldwide, including maize, rice and groundnuts. Dietary exposure to aflatoxins is a public health concern due to their carcinogenic, acute and chronic effects. Rice is an important staple food consumed widely and consists of a major part of the diets for half of the world population. Human exposure to these mycotoxins is a serious problem especially in developing countries where hot and humid climates favor the fungal growth and where food storage conditions are poor and lack of regulatory limits enforcement. The recent developments of biomarkers have provided opportunities in assessing aflatoxins exposure and related health effects in the high-risk population groups. This review describes the worldwide occurrence of aflatoxins in rice during the period from 1990 to 2015 and biomarkers-based evidence for human exposure to aflatoxins and their adverse health effects. Aflatoxin is a potent hepatocarcinogen and humans may expose to it at any stage of life. Epidemiological studies reported an association between aflatoxin intake and the incidence of hepatocellular carcinoma in some sub-Saharan and Asian countries. Even daily high intake of rice with a low level of contamination is of health concern. Thus, it is necessary to implement effective strategies to prevent contamination and fungal growth in rice. A good agricultural and manufacturing practice should be applied during handling, storage and distribution of rice to ensure that aflatoxins contamination level is lower in the final product. Moreover, a regular survey for aflatoxins occurrence in rice and biomarkers-based studies is recommended to prevent and reduce the adverse health effects in the world population.

## Highlights

• Conducted a review of the worldwide occurrence of aflatoxins in rice and associated health effects.
• High variability in aflatoxin contamination and detection levels in rice has been observed.
• In some sub-Saharan and Asian countries, humans are exposed to aflatoxins.
• A good agricultural and manufacturing practice may help to reduce the aflatoxin level in rice.
• Human biomonitoring can be an alternative approach to assess human exposure to aflatoxins.

## 1 Introduction

Aflatoxins are a family of toxins produced by *Aspergillus* species (mainly *A. flavus* and *A. parasiticus*) that contaminate cereals and dietary staples, including maize, rice and groundnuts [1,2]. These fungi are widely distributed in agriculture and highly prevalent in tropical regions specifically sub-Saharan Africa and South East Asia, where hot and humid climates favor fungal growth on food commodities [3]. The four major aflatoxins are aflatoxin B_1_ (AFB_1_), aflatoxin B_2_ (AFB_2_), aflatoxin G_1_ (AFG_1_), and aflatoxin G_2_ (AFG_2_). Aflatoxin M_1_ (AFM_1_) is a less toxic metabolite of AFB_1_ produced in farm animals that consume aflatoxins contaminated feed. AFB_1_ is the most occurring one and has been identified as the group-1 hepatocarcinogen in animals and humans [2]. The occurrence of aflatoxins and their metabolites in foodstuffs are a matter of concern in terms of human health and economic interest [4,5].

Rice is the dominant grain after wheat for half of the world population, provides more than 20% of their daily calories [6]. Asia is the leading continent for the production and consumption of rice. In general, rice is cultivated in subtropical environments with hot and humid climates that stimulate the fungal growth and production of secondary metabolites. Rice can be contaminated by aflatoxins producing fungi when the climatic conditions become favorable for their growth in the field, during harvest, handling and storage [7,8]. The occurrence of aflatoxins in rice has been reported in several studies with a high prevalence in Asian countries [8,9]. The high prevalence of aflatoxins contamination in rice and rice products underscore the importance of intensive monitoring of this dietary staple worldwide.

According to the World Health Organization (WHO), aflatoxin is a global food security concern [10]. In considering toxicity and carcinogenicity, the presence of aflatoxin in rice is a serious public health concern especially in developing countries where people are at risk of aflatoxin exposure [11]. Rice and other staple food are susceptible to aflatoxin contamination [12,13]. Low aflatoxin awareness, food insecurity and lack of regulatory limits enforcement are the significant contributors to high aflatoxin exposure of these populations [11].

Recent developments of aflatoxin biomarkers have provided opportunities in assessing aflatoxins exposure and its associated health effects in the high-risk population groups. Biomarkers analysis in human body fluids covers mycotoxin intake from all dietary sources and exposure by various routes [14,15]; thus human biomonitoring may provide valuable insights, especially in developing countries where AFB_1_ contaminant food data are scarce [16]. In this situation, biomarkers analysis in human body fluids might be important tool to estimate the impact of aflatoxins reduction intervention on public health [11]. Several biomarkers-based studies have been conducted in the last decades to assess aflatoxins exposure in humans [17,18]. The presence of aflatoxins in rice attracts worldwide attention because of the significant economic losses associated with their negative impacts on animal and human health and trade [15,19]. Hence, this review aimed to describe the worldwide occurrence of aflatoxins in rice during the period between 1990 and 2015 and biomarkers-based evidence for human exposure to aflatoxins and its associated health effects.

### 1.1 Toxicity of aflatoxins in animals and humans

AFB_1_ is the most prevalent aflatoxin and a potent hepatocarcinogen in various species, including humans and has been classified as a group 1 carcinogen [2]. In mycotoxins research, most of the research that has been conducted focused upon the study of aflatoxin B_1_ due to its strong carcinogenic effects on human beings. The main human cytochrome P450 (CYP) enzymes involved in human AFB_1_ metabolism in the liver are CYP3A4, 3A5 and 1A2 [20]. In AFB_1_ metabolism, diversity has been observed in different animal species [21] and the most critical reaction is bioactivation to (endo-, exo-) AFB_1_-8,9-epoxide, a highly reactive metabolite which covalently binds to DNA and induces mutations or forms adducts with proteins. A recent study indicated that residual AFB_1_ in the liver negatively affects the p53 and protein Rb pathways in hepatocellular carcinoma [22]. Hepatitis also affects aflatoxins exposure in humans. It has been demonstrated that AFB_1_ and hepatitis B virus (HBV) are synergistic causative agents of hepatocellular carcinoma [23]. Infection by HBV directly or indirectly sensitizes hepatocytes to the carcinogenic effects of AFB1 [23]. In an epidemiological study, a higher concentration of AFB_1_ adducts was found in chronically infected Gambian children and adolescents with HBV than uninfected individuals [24].

AFB_1_ possess toxic effects with a range of consequences; large doses cause acute toxicity and death whereas, chronic sublethal doses induce tumors and impair growth [25]. Acute toxicity of AFB_1_ has been well elucidated in animal experiments: the most susceptible species are ducks, and rabbits while chickens and rats have comparatively greater tolerance [25]. AFB_1_-induced hepatotoxicity occurs in a dose-dependent manner in a rat model [26]. The degree of AFB_1_ toxicity depends on age, sex, species, dose as well as nutritional status and length of exposure; young animals are being more sensitive than adults [25,27]. It is important to note that a number of the toxicological studies have been performed in non-realistic high doses and effects under the RLRS (real-life exposure scenario) approach of low doses and exposure of chemical mixtures were lacking [28,29].

There is limited information on acute aflatoxin toxicity in humans. Acute poisoning in humans has been reported in developing countries, for example, the severe acute aflatoxicosis outbreak in Kenya in 2004 with a mortality of 39.4% [30,31]. Abdominal pain, vomiting, fatty liver and necrosis are common acute poisoning in humans [27]. Other symptoms include depression, anorexia, diarrhea, jaundice, and photosensitivity [27]. On the other hand, acute aflatoxicosis is more common in animals, because of highly contaminated feed and the susceptibility of livestock species to this toxin [27]. Chronic aflatoxicosis in animals is associated with weight loss, lower feed conversion, decreased egg or milk production and increased susceptibility to infectious diseases [27]. In human beings, prolonged consumption of aflatoxin-contaminated food has been linked to liver cancer [32], impaired immune function, decreased reproductive functions, visceral encephalopathy and pulmonary interstitial fibrosis [27,33].

### 1.2 Worldwide rice production and consumption

**Fig. 1:**
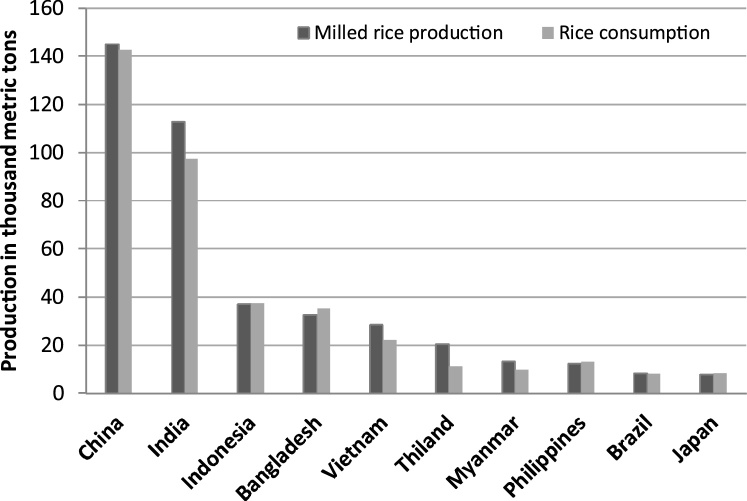
Rice production and consumption in top ten countries in the world in 2017/2018. https://www.statista.com/topics/1443/rice/.

Rice is an important dietary staple that is largely consumed after wheat that consists of a significant part of the diets for half of the world population [34]. Rice is composed of 27% of the global diet and 20% of dietary protein intake in low and middle-income countries [35]. A diverse production system and consumption patterns have been observed for this important food commodity in the world. About 89% of the world’s rice is produced in Asia, with China and India leading the way accounting for 55% of the production [36]. However, it is not equally consumed throughout the country, with more urbanized nations such as Japan experiencing per capita consumption of 65 kg which is four times less than an overpopulated country like Bangladesh (258 kg) [36]. So far, rice is cultivated on 144 million hectares throughout the continent, with China and India dominating with over 50% of the total area harvested and the area under rice cultivation [36]. According to a recent report, nearly 487.5 million metric tons of milled rice were produced in 2017/2018 with a greater production volume in China and India (https://www.statista.com/topics/1443/rice/). The total global consumption of milled rice was approximately 485 million metric tons in that year. According to the above source, the rice consumption in China was about 143 million metric tons and the global use of rice per capita amounted to about 54 kg in that year. The worldwide top ten countries of rice production and consumption are depicted in Fig. 1. In America, maximum rice is produced in Brazil; in Africa, Egypt and Nigeria are the leading rice producer [37]; and in Europe, it is mainly produced in France and Spain [38].

### 1.3 Worldwide occurrence of aflatoxins in rice

The worldwide occurrence of aflatoxins in rice is presented in Table 1. The presence of aflatoxins in rice is relatively high in tropical and subtropical regions in the world, where climatic conditions provide an optimal environment for fungal growth on food and feed [39]. Rice is generally cultivated in flooded irrigation conditions and high moisture levels that favor mold growth and subsequent mycotoxin contamination [34,40]. Among several mycotoxins, the most potent carcinogenic mycotoxins are aflatoxins which are mainly produced by *Aspergillus flavus, Aspergillus parasiticus* and the rare *Aspaergillus nomius* [41]. These fungi can grow on rice under favorable conditions such as floods and heavy rainfall during harvest and storage. Insufficient sun-drying and inappropriate storage make the rice prone to fungal attacks [40]. The contamination level of aflatoxins in rice varies from continent to continent. Several studies reported the occurrence of aflatoxins in rice from different continents.

**Table 1:** Occurrence of aflatoxins in rice in Asia.from 1990 to 2015.

Country, survey year	Type of rice	Origin of rice	Aflatoxins	Analytical Method	LOD/LOQ (μg/kg)	Incidence n (%)	Range (μg/kg)	Mean (μg/kg)	Reference
**ASIA**									
Bangladesh	Rice	Markets	AFB_1_	HPLC	0.2/0.5	–	<LOD-0.9	0.3	[131]
China	Rice	Stores, granaries, markets	AFB_1_	DLLME-HPLC	0.009/0.03	235/370 (63.5)	0.03-20.0	0.06 ± 2.1	[42]
			AFB_2_		0.006/0.02	65/370 (18)	nd-1.6	0.15 ± 0.28	
			AFt			235/370 (63.5)	0.03-21.0	0.65 ± 2.3	
China	Rice	Household	AFB_1_	ELISA	0.01/-	29/29 (100)	0.1-1.4	0.5-0.6	[43]
China	Rice	Local markets	AFB_1_	HPLC-FD	0.012/-	16/84 (19)	0.15-3.22	–	[44]
India	Parboiled rice	Markets	AFB_1_	HPTLC	5.0/-	581/1511 (38.5)	<LOD-361	–	[45]
India	Paddy rice and milled rice	Markets	AFB_1_	ELISA	0.02ppt/-	814/1200 (67.8)	0.1-308	–	[46]
Indonesia	Rice products	Supermarket, traditional market	AFB_1_	ELISA	–	2/2 (100)	2.0-7.0	–	[47]
Iran	Rice	Markets	AFB_1_	IAC, HPLC-FD	0.008/0.025	27/30 (90)	<LOQ-15.15	2.9 ± 4.4	[48]
Iran	Rice	Local markets	AFB_1_	LC-MS/MS	-/0.3	14/65 (21.5)	<LOQ-30.83	3.90	[50]
			AFB_2_		-/0.6	2/65 (3.1)	0.6-1.26	0.93	
Iran, 2007-2008	Polished rice	Retail markets	AFB_1_	IAC, HPLC-FD	0.01/0.03	251/256 (98)	nd-5.8	1.4 ± 1.0	[51]
			AFt				0.1-6.3	1.6 ± 1.1	
Iran	Rice	Local area	AFB_1_	IAC, HPLC-FD	0.2/0.6	180/261 (69)	0.2-4.3	0.72 ± 0.73	[49]
Korea, 2002	Polished rice	Grocery markets	AFB_1_	ELISA	–	5/88 (6)	1.8-7.3	4.3	[8]
Malaysia	Red Rice	Shops	AFt	IAC, ELISA	–	46/50 (92)	0.6-77.3	14.7 ± 16.2	[52]
Malaysia	Rice based	Supermarkets	AFB_1_	ELISA	0.2/0.35	9/13 (69.2)	0.68-3.79	1.75	[54]
Malaysia	Rice	Retail markets	AFt	HPLC	–	–	3.7-96.3	–	[53]
Pakistan	Rice	Vendors	AFB_1_	TLC	1.0/-	250/262 (95.4)	10.07-24.6	3.80	[56]
			AFB_2_		0.5/-	20/262 (7.6)	0.52-2.62	0.09	
			AFt			250/262 (95.4)	10.07-27.27	3.89	
Pakistan	SK basmati rice	City areas	AFB_1_	HPLC-UV	0.05/0.10	-/361 (13.3)	1.1-32.9	–	[57]
	Basmati rice		AFB_1_			-/585 (18.3)	1.0-15.4	–	
	Parboiled rice		AFB1			-/70 (42.9)	1.1-9.2	–	
	Broken rice		AFB_1_			-/11 (36.4)	2.1-25.3	–	
Pakistan	Brwon rice	Export areas	AFB_1_	HPLC-UV	0.10/-	105/200 (52)	–	0.56	[58]
	White rice					80/200 (40)	–	0.49	
	Parboiled rice					70/119 (59)	–	0.73	
Pakistan	Rice	Retail markets, local industries	AFB_1_	IAC, HPLC-FD	0.05/-	38/68 (56)	–	8.23	[60]
			AFt			38/68 (56)	–	19.54	
Pakistan	Rice and rice products	Local markets, shops, super stores	AFB_1_	IAC, HPLC-FD	0.04/0.20	73/208 (35)	0.04-7.4	2.40 ± 0.43	[55]
			AFt				0.04-7.4	2.40 ± 0.43	
Pakistan, 2013	Rice	Local markets	AFB_1_	IAC, HPLC-FD	0.03/0.12	100/120 (83)	0.21-10.54	3.56	[83]
			AFt		0.14/0.38	100/120 (83)	0.21-11.89	3.79	
			AFB_1_	LC-MS/MS	0.02/0.06	104/120 (87)	0.10-10.88	3.73	
			AFt		0.09/0.24	104/120 (87)	0.10-12.39	3.89	
Pakistan, 2010	Rice	Retail markets, agriculture fields	AFt	HPLC-FD	0.04/0.12	185/413 (45)	LOD-68.3	11.2 ± 3.91	[59]
Philippines, 2003	Brown and polished rice	Rice mill	AFB_1_	IAC, HPLC	–	74/78 (95)	nd-8.55	1.48	[34]
			AFB2		–	74/78 (95)	nd-0.33	0.08	
			AFG_1_		–	74/78 (95)	nd-0.93	0.08	
			AFt		0.025/-	74/78 (95)	nd-8.66	1.53	
SriLanka	Parboiled rice	Mills	AFB_1_	TLC and UV-FD	–	-/485	nd-185	–	[62]
Thailand, 2012-2013	Rice	Markets, retail shops	AFB_1_	IAC, HPLC-FD	0.09/-	83/240 (35)	<LOD-26.61	–	[63]
Turkey, 2006	Rice	Fields	AFt	ELISA	1.0/-	56/100 (56)	nd-21.4	–	[39]
			AFB_1_			58/100 (58)	nd-17.2	–	
Vietnam	Rice	–	AFB_1_	HPLC-FD	0.07/0.22	51/100 (51)	nd-29.8	3.31	[61]
United Arab Emirates	Grain rice	household	AFB_1_	HPLC-FD	–	241/500 (48)	1.2-16.5	–	[64]

DLLME-HPLC: Dispersive liquid-liquid microextraction coupled to high performance liquid chromatography with fluorescence detection, IAC: Immunoaffinity column, SPE: Solid phase extraction, nd: not detectable, LOD: limit of detection, LOQ: limit of quantification.

AFt: Aflatoxins total (ie. sum of AFB_1_, AFB_2_, AFG_1_, AFG_2_), SK: Super Kernel.

#### 1.3.1 Asia

In China, AFB_1_ was found in 235 of 370 samples with an average of 0.06 μg/kg [42]. In another study, AFB_1_ detected in all 29 samples with an average contamination level of around 0.5-0.6 μg/kg [43]. A previous study reported the presence of AFB_1_ in 16 out of 84 samples with a range between 0.15–3.22 μg/kg [44]. The presence of aflatoxins has been reported in rice from India. A study covering rice from 12 states of India reported that about 38.5% out of 1511 samples were contaminated by AFB_1_ [45]. Another survey covered 20 states of India, reported that AFB_1_ was present in 814 of 1200 samples ranging from 0.1 to 308 μg/kg [46]. In Indonesia, AFB_1_ was detected in 2 of 2 rice samples with a range between 2.0–7 0.0 μg/kg [47]. Some studies have been conducted in Iran to monitor aflatoxin in rice and rice products. A recent study reported the presence of AFB_1_ in 27 of 30 samples with an average level of 2.9 μg/kg (range < LOQ-15.15 μg/kg) in Iran [48]. AFB_1_ also detected in rice samples at different levels in Iran [[49], [50], [51]]. In South Korea, AFB_1_ was present in 5 of 88 samples at the range of 1.8–7.3 (mean 4.3 μg/kg) [8]. In Malaysia, rice samples were contaminated with total aflatoxins ie. the sum of AFB_1_, AFB_2_, AFG_1_ and AFG_2_, (ranging from 0.6 to 77.3 μg/kg [52]. The presence of aflatoxins in rice in Malaysia has also been reported in previous studies [53,54].

Several studies have been conducted in Pakistan to analyses the levels of aflatoxins in rice. In a recent study, contamination was found in 73 of 208 samples with AFB1 at the range of 0.04–7.4 μg/kg [55]. The contamination of rice with aflatoxins at different ranges has also been reported in previous studies in Pakistan [[56], [57], [58], [59], [60]]. In the Philippines, AFB_1_ was found in 74 of 78 samples with an average level of 1.48 μg/kg [34] and it was found to be comparatively higher (51 of 100) with an average of 3.31 μg/kg [61]. Aflatoxin contamination was found in rice from Sri Lanka [62]. In Turkey, 58 of the 100 samples were found to be contaminated with AFB_1_ at a range between non-detectable (nd) to 17.2 μg/kg [39]. In Thailand, AFB_1_ was detected in 83 of 240 samples at the range of < LOD-26.61 μg/kg [63]. AFB_1_ was detected in 241 of 500 samples from the United Arab Emirates with a range between 1.2–16.5 μg/kg [64].

#### 1.3.2 Africa

In West Africa, AFB_1_ has been detected in all samples with an average level of 37.2 μg/kg (range 4.1–309.0 μg/kg) in Nigeria [37]. In the Ivory Coast, AFB_1_ has been detected in all rice samples at the range of <1.5–10.0 μg/kg [65]. AFB_1_ has also been reported in rice ranging from LOD to 11.0 μg/kg in Egypt [66].

#### 1.3.3 Europe

Regulation for aflatoxins in food commodities is maintained in a better way in Europe than other continents. Up to now, a few studies have been conducted in Europe to analyse aflatoxins in rice. The occurrence of aflatoxins has been reported in rice from Austria, Scotland, Sweden and Spain. In Austria, aflatoxin B_1_ was detected in 24 samples out of 81 rice samples (range 0.45–9.86 μg/kg) imported mainly from Asian countries [67]. In Scotland, 1 out of 33 rice samples (Asian origin) has shown contamination with a mean 14.7 μg/kg of total aflatoxin [68]. Aflatoxin contamination in rice (collected from the Swedish retail market) has been reported in Sweden with a range between 0.1–50.7 μg/kg for total aflatoxin [69]. In Spain, the contamination level was higher in both local and imported samples; aflatoxin total was detected in almost all samples with an average concentration of 37.3 μg/kg (range 1.6–138.3 μg/kg) [38].

#### 1.3.4 America

In Canada, AFB_1_ in rice (imported from Asia and the United States) was found in 99 of 200 samples with a mean level of 0.34–0.39 μg/kg [70]. The contamination was higher in Brazil, of 230 samples, 135 were contaminated with total aflatoxin with an average level of 13.3 μg/kg [71]. In Colombia, AFB_1_ was detected in 4 of 40 samples with a mean level of 7.1 μg/kg [72]. The detection frequency of AFB_1_ was lower (3 of 43) in paddy rice from Ecuador with a mean level of 20. 6 μg/kg [73]. In Mexico, aflatoxin total was found in rice imported from Asian countries with an average level of 16.9 μg/kg [38].

### 1.4 Methods and approaches in aflatoxins analysis

Aflatoxins in rice can be measured by applying different analytical methods (see Table 1, Table 2). The widely used methods are high-performance liquid chromatography with fluorescence detection (HPLC-FD) and enzyme-linked immunosorbent assay (ELISA) [39,74]. ELISA is a largely used technique over HPLC-FD and TLC methods due to its high throughput and requires low sample volumes, minimal sample extraction and clean-up process [39]. This method is rapid, simple and specific and can be used for the quantitative purpose for the detection of mycotoxins in food and feeds in the field [39]. However, this technique often overestimates the targeted analyte in the samples because of the cross-reactive nature of antibodies with compounds similar to mycotoxins. The ELISA test kit has been validated and applied for the detection of total aflatoxins in milled rice with the comparison of HPLC-FD [75]. This technique is often used for screening purposes in highly contaminated samples and also in biomarker-based epidemiological studies. On the other hand, sample clean-up and enrichment of the analyte by immunoaffinity columns and HPLC-FD analysis is more sensitive and reliable for measuring of aflatoxins in food and biological specimens. Although some studies validated the accuracy of ELISA against a reference method applying HPLC-FLD and reported a good correlation between HPLC and ELISA when the same extracts were used [39].

**Table 2:** Occurrence of aflatoxins in rice in Africa, America and Europe.from 1990 to 2015.

Country, survey year	Type of rice	Origin of rice	Aflatoxins	Analytical Method	LOD/LOQ (μg/kg)	Incidence n (%)	Range (μg/kg)	Mean (μg/kg)	Reference
**AFRICA**									
Egypt	Rice grains	Local markets	AFB_1_	IAC, HPLC-FD	0.01/-	-/40	nd-11.0	–	[66]
			AFt				nd-21.7	–	
Nigeria	Rice	Fields, storage, markets	AFB_1_	HPLC	0.02	21/21 (100)	4.1–309.0	37.2 ± 14.0	[37]
			AFB_2_		0.01	21/21 (100)	1.3–24.2	8.3 ± 1.1	
			AFG_1_		0.01	21/21 (100)	5.5–76.8	22.1 ± 3.4	
			AFG_2_		0.06	19/21 (90)	3.6–44.4	14.7 ± 2.5	
			AFt				27.7–371.9	82.5 ± 16.9	
Ivory Coast	Rice	Markets	AFB_1_	ELISA	–	10/10 (100)	<1.5-10.0	–	[65]
**AMERICA**									
Brazil, 2007-2009	Rice	Rice mills	AFB_1_	IAC, HPLC-FD	0.03/-	128/230 (55.7)	0.08-180.7	9.1	[71]
			AFt		0.01-0.03/-	135/230 (58.7)	0.11-207	13.3	
Canada, 2008-2009	Rice	Markets	AFB_1_	IAC, HPLC-FD	0.002/0.05	99/199 (49.7)	nd-7.1	0.36 0.18	[70]
Colombia	Rice and rice products	Supermarkets, Retails stores, Stock centres	AFB_1_	LC-FD	1.0	4/40 (10)	nd-13.6	7.1	[72]
Ecuador	Paddy rice	Rice mills	AFB_1_	IAC, UHPLC/TOFMS	4.0/8.0	3/43 (6.9)	4.9-47.4	20.6	[73]
			AFG_1_		7.0/14.0	1/43 (2.3)	63.7	–	
			AFG_2_		3.0/5.0	1/43 (2.3)	3.3	–	
	Polished rice		AFG_1_		1.0/1.0	1/46 (2.2)	2.0	–	
Mexico, 2008-2009	Rice	Local stores Supermarkets	AFt	IAC, HPLC-FD	0.4-0.6/ 1.2-1.9	-/67	–	16.9	[38]
**EUROPE**									
Austria	Rice	Market	AFB_1_	IAC, HPLC-FD	0.1/0.5	24/81 (29.6)	0.45-9.86	–	[67]
			AFB_2_		0.15/0.5		1.5	–	
Scotland	Rice	Retail market	AFt	SPE, HPLC-FD	–	1/3 (33.3)	0.4-14.7	14.7	[68]
Spain	Rice	Local stores Supermarkets	AFt	IAC, HPLC-FD	0.4-0.6/ 1.2-1.9	-/67	1.6-138.3	37.3	[38]
Sweden	Rice	Retail market	AFt	IAC, HPLC-FD	-/0.1	-/99	nd-50.7	–	[69]
			AFB_1_				nd-46.2	–	
			AFB_2_				nd-4.5	–	

DLLME-HPLC: Dispersive liquid-liquid microextraction coupled to high performance liquid chromatography with fluorescence detection, IAC: Immunoaffinity column, SPE: Solid phase extraction, nd: not detectable, LOD: limit of detection, LOQ: limit of quantification.

AFt: Aflatoxins total (ie. sum of AFB_1_, AFB_2_, AFG_1_, AFG_2_), SK: Super Kernel.

### 1.5 Dietary intake and consumer health

Dietary daily intake of aflatoxins depends on the levels in the food and the amount of food ingested [76]. Due to genotoxic and carcinogenic properties of aflatoxins, the tolerable daily intake (TDI) cannot be considered as a safety factor, so human exposure should be reduced to levels as low as possible [39]. However, a provisional maximum tolerable daily intake (PMTDI) of 1 ng AFB_1_ per kg body weight per day for adults and children without hepatitis B and 0.4 ng AFB_1_ per kg body weight per day for adults with hepatitis B may be used as a guide value in the risk assessment of AFB_1_ from food [77]. An association between dietary aflatoxins exposure and the incidence of human liver cancer has been reported in some African and Asian countries [78]. To make awareness, many countries have set the maximum levels of aflatoxins in foodstuffs as a safeguard of human health, as well as the economic interest of crop producers and traders [79]. The maximum tolerable limit of aflatoxin in rice set by different countries and regulatory bodies are presented in Table 3. In order to protect public health, the European Union has set a maximum level of aflatoxin B_1_ and total aflatoxins (2 μg/kg and 4 μg/kg, respectively) in rice desired for human intake [80], while [81] set maximum levels of aflatoxin B_1_ and total aflatoxins (5 μg/kg, 10 μg/kg, respectively) in rice before human consumption. A comparable regulatory limit for total aflatoxin has been reported in India (30 μg/kg), Brazil (30 μg/kg) Mexico (20 μg/kg), USA (20 μg/kg), Canada (15 μg/kg) Taiwan (10 μg/kg) [6]. In Japan, Korea and China the reported regulatory limit for AFB1 is 10 μg/kg [6]. The lowest regulatory limit for AFB_1_ (1 μg/kg in) has been reported in Bosnia and Herzegovina [82] and Switzerland [41].

**Table 3:** Maximum residual limits (MRLs) of aflatoxin in rice in EU and other countries.

Countries/ Organization	Aflatoxin	MRLs (μg/kg)	Reference
Bosnia and Herzegovina	AFB_1_	1	[82]
Brazil	AFB_1_	30	[6]
Canada	AFt	15	[6]
Chile	AFt	5	[82]
China	AFB_1_	10	[6]
Egypt	AFt	5	[6]
EU	AFB_1_	2	[132]
India	AFt	30	[6]
Iran	AFB_1_	5	[49]
Japan	AFB_1_	10	[6]
Korea	AFB_1_	10	[6]
Malyasia	AFt	5	[52]
Mexico	AFt	20	[6]
Russia	AFB_1_	5	[6]
Switzerland	AFB_1_	1	[41]
Taiwan	Aft	10	[6]
Turkey	AFB_1_	2	[6]
USA	Aft	20	[6]

EU, European Union; AFt, Aflatoxin total.

### 1.6 Exceeding the regulatory limit in rice

Several studies reported the levels of aflatoxins in rice that exceeded the recommended limit value (Table 4). In a recent study in China, 5 of 370 samples (1.4%) exceeded the maximum regulatory limit for AFB_1_ [42]. In India, 256 of 1511 (17%) samples were found to exceed the AFB_1_ regulatory limit [45]. In another study, 24 of 1200 samples (2%) had AFB_1_ levels above the regulatory limit [46]. In Iran, 55 of 256 polished rice (21%) were found to exceed the maximum limit for AFB_1_ [51]. Another study in the same country showed that 3 of 63 (4.6%) samples had the AFB_1_ concentration above the regulatory limit [50]. In Malaysian red rice, 35 of 46 samples (70%) showed the total aflatoxin concentration above the maximum limit [52]. Several studies conducted in Pakistan reported the AFB_1_ contamination levels in rice above the regulatory limit. In a recent study, 19 out of 208 samples exceeded the recommended limit [55]. The other studies also reported the close percentage of samples that exceeded the maximum limit [57,58,60,83]. In Thailand, 12 of 240 samples (5%) had AFB_1_ levels above the regulatory limit [63]. In Vietnam, 10% rice (10 of 100) exceeded the maximum limit for AFB_1_ [61]. In Turkey, 32% of the rice exceeded the maximum tolerable limit for total aflatoxins and 14% of rice samples exceeded this limit for AFB_1_ [39]. In a study in Sri Lanka, aflatoxin levels in parboiled rice were found to be several times higher than TDI with the highest levels of AFB_1_ being 185 μg/kg [62]. In Austria, 3 of 81 samples (3.7%) imported mainly from Asian countries had the AFB_1_ levels that crossed the maximum limit [67].

**Table 4:** Exceeded maximum tolerable limit of aflatoxin in rice of different countries.

Countries	Type of rice	Sample (n)	Aflatoxin	Limit (μg/kg)	Exceeded, n (%)	Reference
China	Rice	370	AFB_1_	2	5 (1.4)	[42]
India	Parboiled rice	1511	AFB_1_	30	256 (17.0)	[45]
India	Paddy rice	1200	AFB_1_	30	24 (2.0)	[46]
Iran	Rice flour	30	AFB_1_	0.1^a^	20 (67)	[48]
Iran	Polished rice	256	AFB_1_	2	55 (21)	[51]
			AFt	4	7 (2.7)	
Iran	Rice	63	AFB_1_	5	3 (4.6)	[50]
Malaysia	Red rice	46	AFt	5	35 (70)	[52]
Pakistan	SK Basmati rice	361	AFB_1_	2	(6.4)	[57]
Pakistan	Brown rice	200	AFB_1_	2	(5.6)	[58]
Pakistan	Rice	68	AFB_1_	2	18	[60]
Pakistan	Rice and rice products	208	AFB_1_	2	19	[55]
Pakistan	Rice	120	AFB_1_	2	44	[83]
Thailand	Rice	240	AFB_1_	2	12 (5)	[63]
Vietnam	Rice	100	AFB_1_	2	10 (10)	[61]
Turkey	Rice	100	AFt	4	32 (32)	[39]
Austria	Rice	81	AFB_1_	2	3 (3.7)	[67]

a 0.1 μg/kg: maximum established level of EU regulations for baby food. AFt, Aflatoxin total.

### 1.7 Aflatoxins exposure and health effects

Human exposure to aflatoxins occurs through the consumption of contaminated foodstuffs and such exposure can be happened throughout the life course, beginning in utero via transplacental exposure [84]. Human breast milk is one of the major pathways of aflatoxin exposure for young children during the breastfeeding period [85]. Consumption of AFB_1_- contaminated food might result in the secretion and presence of AFM_1_ (a metabolite of AFB_1_) in human breast milk. Therefore, children’s exposure to AFM_1_ through breastfeeding is at high risk for the life-threatening side effects of aflatoxins. Preliminary evidence suggests an interaction between chronic aflatoxin exposure and malnutrition, as reduced uptake of nutrients from the diet, may result in growth retardation in children [86]. An association between aflatoxin exposure in utero and growth faltering has been reported in Gambian children [87]. Chronic aflatoxin exposure is linked with kwashiorkor, a severe Protein Energy Malnutrition (PEM) disease [88]. Studies conducted in the last three decades have shown a higher aflatoxin concentration in the blood and urine of children with kwashiorkor compared to healthy children [89]. Besides growth impairment, chronic aflatoxin exposure also affects the immune system. A decreased IgA was found in the saliva of children who were highly exposed to AFB_1_ [90].

Acute aflatoxicosis in humans occurs during high exposure over a relatively short time. For example, in Kenya in 2004, 317 individuals were diagnosed with acute liver failure of which 37% subsequently died as a result of acute aflatoxicosis [91]. Chronic aflatoxicosis occurs because of low dose aflatoxin exposure over a long period which is more prevalent than acute aflatoxicosis. Liver cancer is the well-known health effects of chronic aflatoxicosis in human and it was the sixth most common cancer worldwide in 2012, with over 80% of cases in developing countries in Africa and Asia [11]. Both aflatoxin and hepatitis B exposure have been reported in epidemiological studies, resulting in an increased risk of hepatocellular carcinoma in these countries [92,93]. Aflatoxins have also been found to be linked with other liver diseases such as cirrhosis and hepatomegaly [11]. In Asia, an outbreak of hepatitis due to aflatoxin was reported in the states of Rajasthan and Gujrat in India, resulting in an approximate 106 deaths in 1974 [94]. Another outbreak of aflatoxin affecting both humans and dogs was reported in northwest India in 1974 [95]. Avoiding contaminated diet, agricultural reforms with changing more aflatoxins susceptible crops into less susceptible crops and implementation of the hepatitis B virus immunization program may have the potential in reducing and preventing hepatocellular carcinoma in humans.

### 1.8 Aflatoxin biomarkers measurement in biological fluids

Aflatoxin biomarkers have been established and validated in epidemiological studies that investigated the association between exposure and risk of diseases [96,97]. Various analytical techniques such as ELISA and HPLC-FD have been widely used in aflatoxins biomarkers analysis in human body fluids. LC–MS/MS multitoxin approach has also been applied in aflatoxin biomarker analysis in human urine [98,99]. Among these techniques, HPLC-FD was found to be a more specific and sensitive one for aflatoxin biomarker studies [16]. Such investigations have analyzed the levels of serum AF-alb or AFB_1_-lysine adduct in blood or AFB_1_-N_7_-guanine and AFM_1_ metabolite levels in urine as valid biomarkers of aflatoxins exposure. Human biomonitoring is an effective tool and may provide valuable insights, especially in developing countries where food contaminated data are scare or no regular surveillance of mycotoxins exists [16]. Mycotoxin biomarkers analysis in human body fluids covers mycotoxin intake from all dietary sources and exposure by several routes [100]. The concentration of AFB_1_-lysine albumin in serum indicates exposure over a period of several weeks or months because of its long half-life in blood, whilst AFB_1_-N^7^-guanine or AFM_1_ in urine reflects recent exposure and thus used as a short-term biomarker of exposure [18,101,102]. In the last decades, several studies reported the presence of aflatoxin biomarkers in human body fluids. High human exposure was found in rural subsistence farming communities in developing countries especially in Asia and Africa [11]. Aflatoxin exposure has been rarely reported in developed countries as strict regulation for aflatoxins in food is maintained there. An early study reported a correlation between daily intake of AFB_1_ and its urinary excretion of AFM_1_ [103].

#### 1.8.1 AF-alb and AFB1-lysine adducts in blood

In East African countries, analysis of AF-alb biomarker in serum indicated a high aflatoxin exposure, where it was detected in 78% out of 597 serum samples (range ND-211 pg/mg) from Kenya [104], in Uganda, this biomarker was detected in 98% (192 of 196) samples (range ND-238 pg/mg) [105], in Tanzania, where AF-alb was detected in 67–99% of samples collected from children and adults [106]. In the North and South part of Africa, the prevalence of exposure was comparatively lower than the levels found in East and West Africa. AF-alb was found in 67% of 46 samples (range ND-32.8 pg/mg) from Egypt [107]. AF-alb was detected in 35% (34 of 98) of serum samples from pregnant women in Egypt [108]. In Asia, AFB_1_-lysine adduct was detected in 97% of 170 samples (range 0.2–23.3 pg/mg) from Malaysia [109]. A recent study [101] reported the presence of AFB_1_-lysine biomarkers in 94% of 141 samples (range 0.4–2939.3 pg/mg) from pregnant women in Nepal. In the same investigation, this biomarker was detected in 100% (63 of 63) samples from pregnant women from Bangladesh as well as in 100% (63 of 63) cord blood samples and in infants 100% (63 of 63) whose mothers were exposed to aflatoxin during pregnancy. In Brazil, AFB_1_-lysine adduct was found in 62% samples in a concentration ranging from ND to 57.3 pg AFB_1_-lysine/mg blood albumin [110].

#### 1.8.2 AFM1 biomarkers in urine

Compared to blood, urine has widely used a matrix for AFM_1_ biomarker analysis because of its non-invasive sampling and better acceptance by the participants in field studies [16,111]. In low-income countries where food contaminated data are scarce, biomonitoring may be an effective tool to gain more insights into human exposure to aflatoxins. Recently, many have been conducted to analyse the AFM_1_ biomarkers in humans of different countries [16].

In Asia, AFM_1_ biomarker has been frequently detected in urines from Malaysia, China, and Bangladesh. AFM_1_ was detected in 98 of 160 urines (61%) in a concentration ranging from LOD-74.7 pg/mL (mean 23.4 pg/mL) in a Malaysian cohort [112]. A recent study in the Zhejiang province of China indicated the presence of AFM_1_ in urines from adult males (mean of 51.5 and range LOD–4900 pg/mL) and pregnant women cohort (mean of 50.3 and range LOD-3500 pg/mL) [113]. Another study in this country, reported far more frequent detection and higher AFM_1_ level in 1988 (mean 48 and range 5.7–243 pg/mL) than in 2000 (only one urine 9 pg/mL) [114]. In a recent study in Bangladesh, AFM_1_ was detected in 40% of urine samples (mean 13.6 and range of 1.7–104 pg/mL) in summer and 42% of samples (mean 27.7 and range of 1.8–190 pg/mL) in the winter season [16]. In the same study, AFM_1_ was detected in 17 of 54 (31%) urines (mean 13.9 and range 1.7–141.5 pg/mL) from pregnant women cohort in Bangladesh. AFM_1_ was present in 3 of 60 urines from Thailand, (range 160–550 pg/mL), [115].

In Africa, the highest AFM_1_ levels in urines were found in Ghanian adults (mean 1800, range LOD–11562 pg/mg creatinine; [116]. A recent study in Nigeria reported AFM_1_ occurrence in children and adolescents urines (mean 300 and range LOD–1500 pg/mL; [117] and urines from Guinean infants (mean 97 and range 8–801 pg/mL [118]; reveal that AFB_1_ exposure is a serious health concern in several sub-Saharan African countries. Another study detected urinary AFM_1_ in Ivory Coast [119] and Cameroon [120].

In Europe, urines from Germany (n = 30 and n = 50) and Belgium (n = 32) had no detectable levels of AFM_1_ in urines [98,121,122]. In Southern Italy, only 3 of 52 urines had detectable levels of AFM_1_ (mean 68 and range 20–146 pg/mL; [99].

There are a few reports from America. Three studies conducted in Brazil reported the presence of AFM_1_ in urines. A recent study detected AFM_1_ in 65% urine samples (range 0.37 to 1.70 pg/mg creatinine; [123]. Another study reported AFM_1_ occurrence in urines (mean 1.2 pg/mL, range 0.25–6.9 pg/mg creatinine; [124]. The other study conducted in Brazil detected urinary AFM_1_ (5.9 pg/mL range 1.8–39.9 pg/mL; [125]). In the USA, a study in adults with an elevated risk of liver cancer reported urinary AFM_1_ in 11.7% of 179 samples (mean 223.8 pg/mg creatinine, range 1.89–935.49 pg/mg creatinine; [126]. In a more recent study, AFM_1_ was found in 14% and 22% of urines from rural and urban Haitians (max. 700 pg/mL) [127].

#### 1.8.3 AFM_1_ biomarker in human breast milk

Beside human blood and urine, breast milk has also been used for AFM_1_ biomarker analysis in some epidemiological studies. AFM_1_ was found in human breast milk (range 5–3400 pg/mL) of the Arab Emirates [128]. In Brazil, AFM_1_ was detected in 1 of 50 samples at a concentration of 0.02 ng/mL [129]. In a recent study in Iran, AFM_1_ was detected in 157 of 160 samples, with a concentration ranging from 0.3 to 26.7 ng/kg [130].

## 2 Conclusions and recommendations

High variability in aflatoxin contamination has been observed worldwide and the contamination is higher in developing countries where rice constitutes the major nutritional source of the diet. Several biomarker-based studies report human exposure to aflatoxins especially in some sub-Saharan and Asian countries. To minimize aflatoxin contamination, applying effective strategies can be the prevention against fungal growth in rice. Implementation of good agricultural and manufacturing practices during harvesting, storage, and distribution of rice may ensure the lower level of aflatoxin in the final product. Physical, chemical and microbiological approaches can be used to reduce the aflatoxin levels in the rice. Hazard Analysis and Critical Control Point (HACCP) approach may also be a useful food safety strategy to control contamination from field to consumer and so that the level does not cross the limit value recommended by the legislation. Biomarkers-based monitoring is recommended to integrate with conventional food analysis approach to assess aflatoxins exposure and related health effects in the population groups who are at high-risk. Finally, considering the importance of rice as a major part of the human diet, further research is needed to reduce the exposure effects of aflatoxins in humans.

## Conflict of interest

The author declare no conflict of interest.
